# Breath-Triggered Drug Release System for Preterm Neonates

**DOI:** 10.3390/pharmaceutics13050657

**Published:** 2021-05-04

**Authors:** Felix C. Wiegandt, Ulrich P. Froriep, Fabian Müller, Theodor Doll, Andreas Dietzel, Gerhard Pohlmann

**Affiliations:** 1 Division of Translational Biomedical Engineering, Fraunhofer Institute for Toxicology and Experimental Medicine ITEM, 30625 Hannover, Germany; ulrich.froriep@item.fraunhofer.de (U.P.F.); fabian.mueller@item.fraunhofer.de (F.M.); theodor.doll@item.fraunhofer.de (T.D.); 2 Department of Otorhinolaryngology, Hannover Medical School, 30625 Hannover, Germany; 3 Institute of Microtechnology, Technische Universität Braunschweig, 38124 Braunschweig, Germany; a.dietzel@tu-braunschweig.de

**Keywords:** aerosol, breath-triggered drug release, nasal prong, preterm neonate, real-time measurement, surfactant

## Abstract

A major disadvantage of inhalation therapy with continuous drug delivery is the loss of medication during expiration. Developing a breath-triggered drug release system can highly decrease this loss. However, there is currently no breath-triggered drug release directly inside the patient interface (nasal prong) for preterm neonates available due to their high breathing frequency, short inspiration time and low tidal volume. Therefore, a nasal prong with an integrated valve releasing aerosol directly inside the patient interface increasing inhaled aerosol efficiency is desirable. We integrated a miniaturized aerosol valve into a nasal prong, controlled by a double-stroke cylinder. Breathing was simulated using a test lung for preterm neonates on CPAP respiratory support. The inhalation flow served as a trigger signal for the valve, releasing humidified surfactant. Particle detection was performed gravimetrically (filter) and optically (light extinction). The integrated miniaturized aerosol valve enabled breath-triggered drug release inside the patient interface with an aerosol valve response time of <25 ms. By breath-triggered release of the pharmaceutical aerosol as a bolus during inhalation, the inhaled aerosol efficiency was increased by a factor of >4 compared to non-triggered release. This novel nasal prong with integrated valve allows breath-triggered drug release directly inside the nasal prong with short response time.

## 1. Introduction

One of the main disadvantages of inhalative therapy with continuous drug delivery is the waste of drug during exhalation [1,2,3]. With a typical inhalation–exhalation ratio of 1:2 for adults and children [4], and 1:3 for (pre)term neonates [5,6], an amount up to 50% for adults and children, and 75% for preterm neonates of the aerosolized drug is lost a priori.

In contrast, breath-triggered drug release enables patient-individualized aerosol delivery, and thus has the following benefits compared to continuous drug release:

1. As the drug is only released during inhalation, the aforementioned loss during exhalation is theoretically reduced to zero, resulting in higher drug exploitation and enormous cost savings [7,8,9].
2. The aerosol can be variably released as a bolus during the inhalation phase, so that different lung regions can be targeted. A release at the beginning of inspiration targets mainly peripheral lung areas, whereas a release towards the end of inspiration targets mainly central lung areas. This also results in a more time-saving treatment and less drug distribution into the body, reducing side effects [10,11,12,13,14,15,16].

There are several studies on breath-triggered drug delivery for adults and children. By contrast, breath-triggered drug delivery is very challenging for (pre)term neonates and fewer studies have been conducted [17,18]. The breathing patterns of preterm neonates have a pivotal role in that challenge: in contrast to children or adults with a breathing frequency of 22 or 12 breaths/min, a 177 or 500 mL tidal volume and an inhalation time of 0.95 or 1.67 s [4], preterm neonates have a small tidal volume of 4–5 mL/kg, a high breathing frequency of 40–50 breaths/min and a short inhalation time of 0.2–0.35 s [6,19]. In addition, preterm neonates with lung pathologies, such as respiratory distress syndrome, for which pulmonary surfactant must be administered, have a further reduced tidal volume and an increased respiratory rate [20,21,22,23,24].

There are numerous systems for breath-triggered drug delivery, such as the AutohalerTM (3M Drug Delivery Systems, St. Paul, MN, USA), the AERx^®^ Pulmonary Drug Delivery System (Hayward, CA, USA), the I-Neb AAD (Philips Respironics, Murrysville, PA, USA) and the AKITA^®^ JET (Vectura Group plc, Chippenham, UK). However, these systems cannot be used for preterm neonates due to their mode of operation and the aforementioned respiratory parameters of preterm neonates. Furthermore, there is the approach of standard aerosolizers, such as the Aerogen^®^ (Aerogen Ltd., Galway, Ireland), to synchronize aerosol production with breathing, feeding the aerosol into the inhalation limb. However, since the distance from the nebulizer to the patient is large, respiration-synchronized aerosol delivery is not possible due to the considerable delay before the aerosol reaches the patient [25].

It has been shown that for the highest possible inhaled aerosol efficiency the aerosol must also be administered as close as possible to the patient interface [25]. The greater the distance between the release of the aerosol into the patient interface, the less efficient the delivery due to the longer aerosol path length and thus increased deposition on the walls and a delay in aerosol arrival [1,26,27,28]. In addition, the aerosol is often fed from the aerosol system into the ventilation circuit via a T-connector [26]. However, the 90° change in flow direction leads to increased particle deposition within the T-connector. Therefore, optimized designs such as the VC connector or the SL design have already been developed and tested for effectiveness. These first prototypes showed an improved efficiency compared to the T-connector [29,30]. However, the aerosol is still not released directly into the patient interface, leading to a reduced inhaled aerosol efficiency as described before.

For the development of a breath-triggered drug release directly inside the patient interface (nasal prong) for preterm neonates, the abovementioned challenges need to be addressed. In this article, we describe a novel nasal prong with an integrated miniaturized aerosol valve with very short response time until full cross-section (<25 ms), releasing the aerosol directly inside the patient interface.

## 2. Materials and Methods

### 2.1. Breath-Triggered Drug Release System

**Figure 1:**
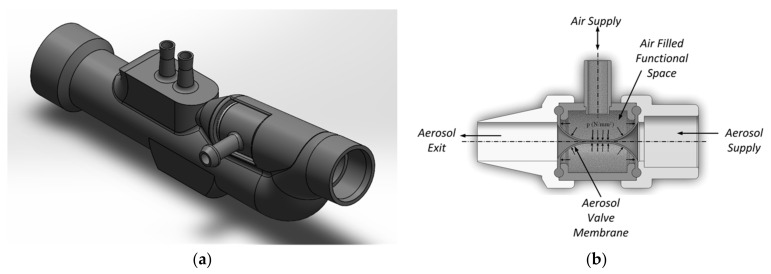
(**a**) Schematic representation of the novel nasal prong with integrated miniaturized aerosol valve and (**b**) the closed aerosol valve’s sectional view in the presence of applied air volume in the functional space.

In this study, we integrated a miniaturized aerosol valve [31] inside a novel nasal prong, enabling breath-triggered drug release directly at the patient interface (Figure 1). This valve contains a silicone membrane (MediTech Franken GmbH, Eckental, Germany) with a shore hardness of 40.

**Figure 2:**
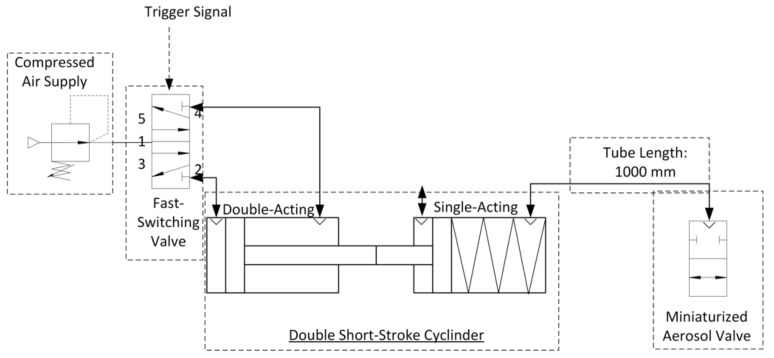
Technical diagram of the aerosol valve control.

This aerosol valve is controlled by a defined air volume of 4 mL provided by a double short-stroke cylinder, which deforms highly elastic silicone membrane in the radial direction. The tube length between aerosol valve and double short-stroke cylinder is 1000 mm (PUN-H-6X1-BL, Festo AG and Co. KG, Esslingen am Neckar, Germany). The advantage of using a double short-stroke cylinder lies in the safety aspect—if the membrane of the aerosol valve is damaged, there is no significant increase in pressure at the patient interface due to the small air volume of 4 mL supplied. The double short-stroke cylinder consists of a single-acting short-stroke cylinder (AEVC-32-5-I-P, Festo AG and Co. KG, Esslingen am Neckar, Germany), controlled by the stroke of the double-acting short-stroke cylinder (ADVC-32-5-I-P, Festo AG and Co. KG, Esslingen am Neckar, Germany). A fast-switching valve (air solenoid valve MHE2-MS1H-5/2-QS-4-K, Festo AG and Co. KG, Esslingen am Neckar, Germany) actuates the double-acting short-stroke cylinder at 6 bar, in accordance with the trigger signal (Figure 2).

The trigger signal is obtained by detecting the inhalation flow of the simulated breathing in the measurement zone “inhaled aerosol” with a mass flow meter (Figure 1) in combination with an analog–digital converter (Labjack U6-Pro, Meilhaus Electronic GmbH, Alling, Germany). As soon as the start of an inhalation phase is detected, the aerosol valve is opened. The three aerosol release modes by controlling the aerosol valve are shown in Table 1.

**Figure 3:**
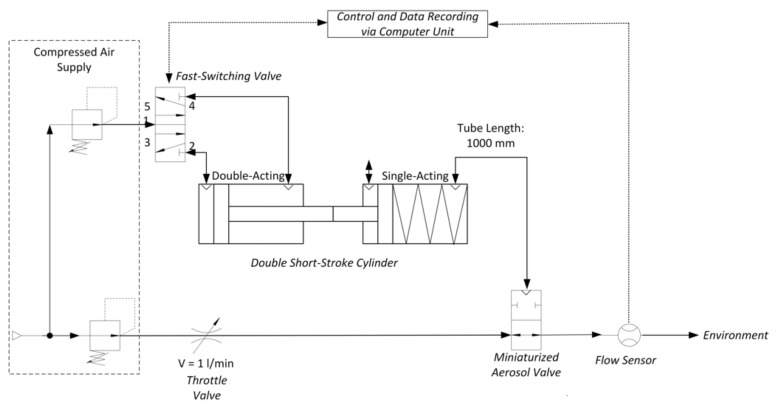
Technical representation of the test bench determining the opening and closing time of the integrated aerosol valve.

To determine the opening and closing time of the aerosol valve integrated in the nasal prong, we used the test bench shown in Figure 3.

In this test bench, a flow rate of 1 L/min is set via a throttle valve, which passes the aerosol valve and is continuously measured by means of a downstream flow sensor (Mass Flow Meter SFM3000, Sensirion AG, Stäfa, Switzerland). The data measured from the flow sensor are recorded by a computer unit. A trigger signal is manually triggered via the computer unit, which, as described above, controls the double short-stroke cylinder via the fast-switching valve and causes the valve to close or open. The time between the two states (open and closed), depending on the detected flow, is measured and serves as a reference value for the speed of the aerosol valve to fully open or close.

### 2.2. Preterm Neonate Test Bench

**Figure 4:**
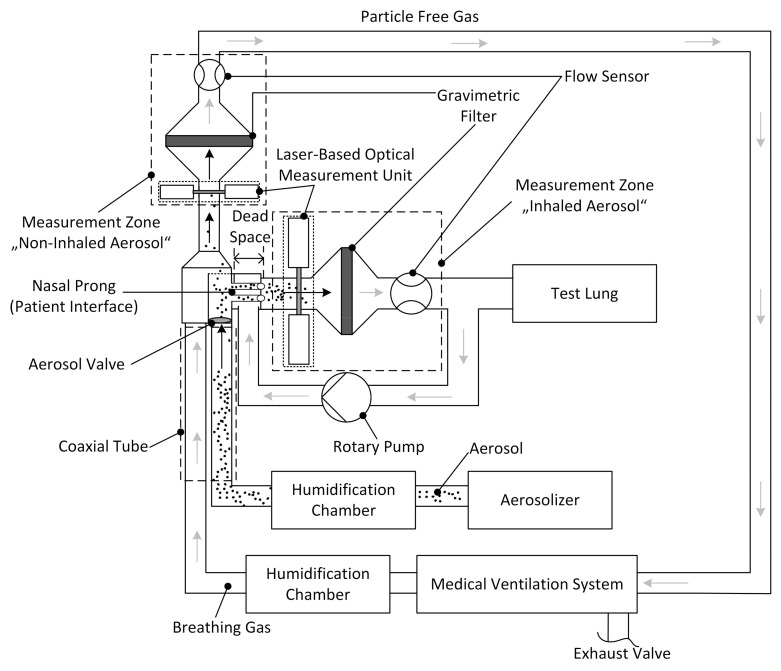
Test bench with two integrated laser-based optical measurement units added upstream from the gravimetric filters, simulating preterm neonate breathing characteristics. This test bench is based on the setup described in Wiegandt et al. [33], Journal of Aerosol Medicine and Pulmonary Drug Delivery, 2021.

In order to be able to determine the aerosol output released by the miniaturized aerosol valve integrated in the nasal prong, we used the preterm neonate test bench (Figure 4) described in Wiegandt et al. [32,33]. In brief, the preterm neonate breathing characteristic was simulated by a test lung, consisting of a silicone bellow and shaft, operated by a linear motor (PS02-23Sx80F-HP-K, NTI AG LinMot and MagSpring, Spreitenbach, Switzerland). A test lung simulated the breathing using an inhalation–exhalation ratio (I:E) of 0.39:0.61, 51 breaths per minute (bpm) and a tidal volume of 12.3 mL. Each measurement cycle was carried out for about 120 s. The medical ventilation circuit was driven by a Babylog^®^ 8000 plus (Drägerwerk AG and Co. KGaA, Lübeck, Germany) and operated in continuous positive airway pressure CPAP-mode with a positive end expiratory pressure (PEEP) of 5 mbar and a breathing gas flow of 6 L/min. The pharmaceutical aerosol was generated by a continuous powder aerosolizer (CPA) system by means of short 10 ms pulses of pressurized gas every 6 s, releasing recombinant surfactant Protein-C (rSP-C) with a median particle size in the range of 3 to 3.5 µm [34,35].

The aerosol, carried by a flow of 0.84 L/min, and the breathing gas were each heated and humidified in the humidification chamber and guided to the patient interface via a coaxial tube.

During a simulated inhalation, the particles were first sucked through the nasal prong (patient interface) into the measurement zone “inhaled aerosol”. Once there, these inhaled aerosol particles were first measured by the laser-based optical measurement unit (light extinction), followed by sampling the particles on the filter. During a simulated exhalation, the remaining non-inhaled aerosol particles were guided backward through the patient interface toward the measurement zone “non-inhaled aerosol”. These non-inhaled particles were then measured by the second laser-based optical measurement unit (light extinction), followed by sampling of these particles on a second filter, resulting in particle-free gas that was conducted back to the medical ventilation system. The total flows $\dot{V}\left(t\right)$ of both measurement zones were each measured with a flow sensor (Mass Flow Meter SFM3000, Sensirion AG, Stäfa, Switzerland). The system was operated under standard laboratory conditions (22 °C and 50% relative humidity); however, it works independently of temperature and humidity conditions.

### 2.3. Aerosol Output Measurements

We determined the aerosol output of each measurement zone gravimetrically and optically. For the determination of dry particle mass, filters with a diameter of 80 mm (glass fiber, Sartorius AG, Goettingen, Germany) were conditioned before (initial weight) and after sampling (final weight). For conditioning, the filters were first dried at a temperature of 60 °C for 6 h, and then kept for 24 h at a constant relative humidity of 30% at room temperature of about 22 °C. Afterward, the filters were weighed three times by an automated measuring robot developed at Fraunhofer ITEM (weighing accuracy ±0.04 mg). The difference between initial and final weight was taken as the mass of dried particles collected on the filter.

For the optical determination, we measured the attenuation of the light beam through the measuring volume (light extinction), as described previously [33]. In brief, we utilized two infrared diode laser systems (Laser Beam Sensor LA-511, Panasonic Electric Works Europe AG, Holzkirchen, Germany) with a sensor beam height of 15 mm, a sensor beam width of 1 mm and operating at a wavelength of 780 nm. The aerosol mass *m_aerosol_* during one measurement cycle (*t*_0_ to *t_end_*) was calculated by the aerosol-specific constant k, the total flow $\dot{V}\left(t\right)$ circulating in the corresponding measurement zone, the attenuated light transmission *I*(*t*) due to the aerosol and the unattenuated initial light transmission *I*_0_ [33]:

(1) $$m_{aerosol}=\frac{1}{k}\int_{t_{0}}^{t_{end}}-ln\left(\frac{I\left(t\right)}{I_{0}\left(t\right)}\right)\dot{V}\left(t\right)\;dt\cdot$$

The aerosol-specific constant k is calculated through a simple linear regression between the gravimetrically weighed dry mass *m_dry_* and $\int_{t_{0}}^{t_{end}}-ln\left(\frac{I\left(t\right)}{I_{0}}\right)\dot{V}\left(t\right)\;dt$ [33]:

(2) $$m_{dry}=\beta_{1}\times \int_{t_{0}}^{t_{end}}-ln\left(\frac{I\left(t\right)}{I_{0}}\right)\dot{V}\left(t\right)\;dt+\beta_{0}\cdot$$

where the regression coefficient *β*_1_ represents the 1/k-factor and the regression coefficient *β*_0_ the water mass *m_water_* [33]. Since *I*_0_ decreases with time in the real system due to aerosol deposition and water condensation on the optical windows, we calculated *I*_0_(*t*) by simple linear regression over the first and last three measurement points of *I*_0_ and the corresponding time *t* as follows [33]:

(3) $$I_{0}\left(t\right)=\beta_{1}t+\beta_{0}\cdot$$

Once the aerosol output was determined, the next step was to determine the efficiency of the breath-triggered drug release system, which could be considered as efficiency of the aerosol valve technology *E_valve_*, neglecting all aerosol losses up to the optical measurement. It can be defined as the ratio of the mass of the inhaled aerosol mass Δ*m_inhaled_* aerosol to the sum of the inhaled aerosol Δ*m_inhaled_* and the non-inhaled aerosol Δ*m_non-inhaled_*:

(4) $$E_{valve}=\frac{\Delta m_{inhaled}}{\Delta m_{inhaled}+\Delta m_{non-inhaled}}$$

## 3. Results and Discussion

In this study we developed a breath-triggered drug release system in which we integrated a miniaturized aerosol valve into a nasal prong proximal to the patient. We characterized the breath-triggered system (nasal prong) using a test bench to determine aerosol output and a test bench to determine the aerosol valve speed. The nasal prong has the following characteristics:

1. Fast opening and closing times of the aerosol valve;
2. Release of an aerosol bolus at defined times;
3. Increase in release efficiency compared to a non-breath triggered system.

In the following sections, we first show that the test bench used can determine pharmaceutical aerosol output optically in real time, via correlation of gravimetric and optical signals, and show the optical signal of aerosol release during non-breath-triggered release. It has been shown that high aerosol losses occur during non-breath-triggered drug release. Therefore, the presentation of a breath-triggered release follows subsequently.

### 3.1. Correlation between Optical and Gravimetric Measurements

**Figure 5:**
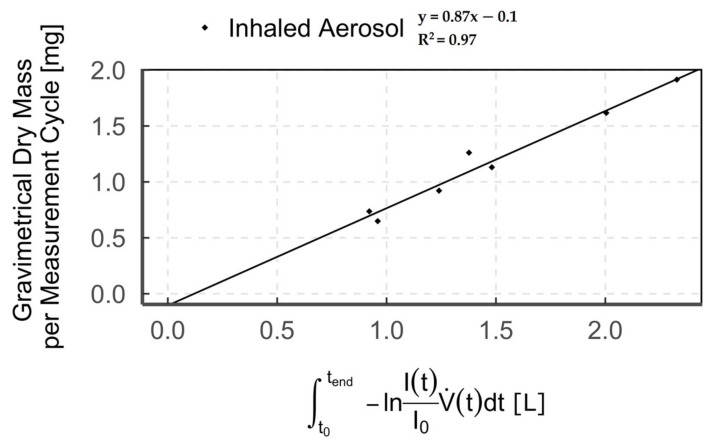
Diagram showing the correlation of optical and gravimetric measurements (points = single measurement cycle) of humidified aerosol for breath-triggered drug release mode 1 in the measurement zone “inhaled aerosol”.

The correlation of the optical measurements with the gravimetrically determined dry mass is shown as an example in Figure 5 for aerosol-triggered mode 1 in the “inhaled aerosol” measurement zone (*n* = 7).

We combined optical (light extinction) with gravimetric (filter) detection to subsequently enable real-time measurement of aerosol output, solely via the measured optical signal. Our results show a high correlation between gravimetric and optical detection of up to R^2^ = 0.97. This high correlation is consistent with data from previous studies [36,37,38,39]. However, we observed that the regression line does not pass through the origin but shows a negative offset on the gravimetric axis. The observed offset results from the comparison between the optical signal of the humidified aerosol and the dry particle mass on the filter [40] and is a measure of the water uptake by the aerosol [33]. This is due to the fact that the particle cross-section increases with water content, depending on the particle’s hygroscopicity [41,42,43,44]. Accordingly, a larger optical signal, due to increased light extinction, is measured for humidified aerosol compared to dry aerosol. Hence, when correlating the optical signal (humidified aerosol) with the gravimetric value (dry aerosol), we obtain the aforementioned offset.

### 3.2. Optical Measurements—Optical Signal

**Figure 6:**
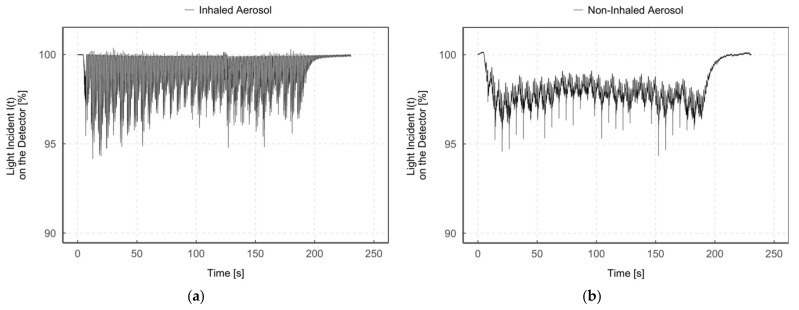
Diagrams of the light incident *I*(*t*) on the detector (%) during time (s) in the measurement zone “inhaled aerosol” (**a**) and “non-inhaled aerosol” (**b**) for humidified aerosol of the same measurement.

Figure 6 shows an example of the light incidence *I*(*t*) on the detector after the aerosol has passed through the measurement zone “inhaled aerosol” (Figure 6a) and “non-inhaled aerosol” (Figure 6b) during continuous aerosol delivery (breath-triggered drug release mode 3).

The light incidence *I*(*t*) on the detector in the “inhaled aerosol” measurement zone (Figure 6a) fluctuates between particle detection (*I*(*t*) < 100%) and no particle detection (*I*(*t*) = 100%), with partial false peak overshoots (*I*(*t*) > 100%). In contrast, a constant signal *I*(*t*) < 100% was detected in the measurement zone “non-inhaled aerosol” (Figure 6b) during the entire measurement period. In addition, both figures simultaneously show periodic narrow bands of signal attenuation due to the operating principle of the CPA with its short 10 ms pulses of compressed gas every 6 s.

Since, as already mentioned, the aerosol can only enter the “inhaled aerosol” measurement zone during the simulation of an inhalation, aerosol is accordingly only detected during that zone. In contrast, it is continuously detected in the “non-inhaled aerosol” measurement zone. The aerosol amount detected in the measurement zone “non-inhaled aerosol” is no longer available for inhalation and is consequently wasted. Thus, with continuous aerosol delivery to the patient interface, aerosol loss mainly depends on the inhalation–exhalation ratio.

**Figure 7:**
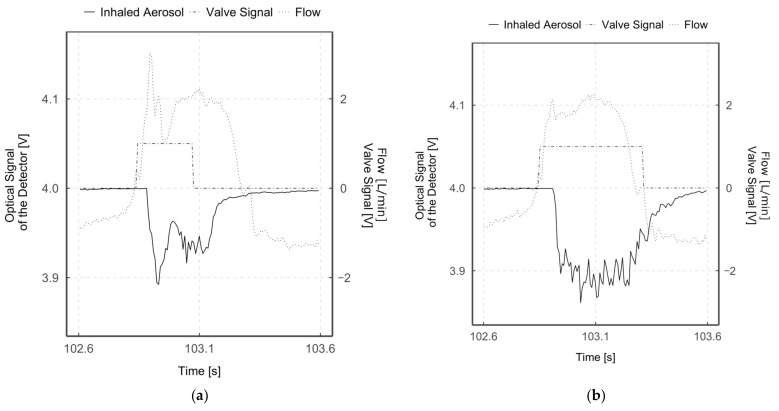
Diagrams of optical signal (%), inhalation flow (L/min) and aerosol valve actuation (V) over time for mode 1 (**a**) and mode 2 (**b**). Optically detected values are normalized to 4V for better overview.

Therefore, we integrated an aerosol valve directly in the patient interface to enable breath-triggered drug release close to the patient. To gain more insight between aerosol valve opening and aerosol detection, we compared single aerosol valve openings with inhalation flow rate and aerosol detection (see Figure 7).

As described, the trigger signal for opening the aerosol valve is triggered when an inhalation phase is detected (starting from negative flow values towards positive ones, intersection at flow = 0 L/min). It can be seen that a delay of around 18 ms occurs between the start of inhalation and the trigger signal being set. This delay is related to the computing power and the execution of the software codes, and the hardware response time of 7 ms.

The valve opening and closing speeds were determined using the test bench described in Section 2.1. The measured time to fully open the aerosol valve was 22.3 ± 1.9 ms and to fully close it was 25 ± 0.5 ms. However, the measured time between aerosol valve opening trigger and optical aerosol detection in the “inhaled aerosol” measuring zone was 44.5 ± 7.3 ms, and the time between aerosol valve closure trigger and almost no aerosol detection was 88 ± 7.6 ms. These optically measured speeds differ from the values measured with the test bench, since the distance of the aerosol from the valve to the optical detection is involved. 

It is noticeable that this detected value for complete aerosol valve opening is faster than complete closing. This is due to the fact that the aerosol valve has to be closed against the spring force of the second short-stroke cylinder and that when the valve is closed, pressure builds up in front of the membrane, which allows it to open more quickly. Conversely, this spring force accelerates the opening of the aerosol valve, which is far more important in the case of breath-triggered drug release, as Longest et al. have shown that the aerosol reaches the deeper airways only during the first half of an inhalation phase [30]. Accordingly, with a typical inhalation duration of 320 ms for a preterm neonate, only during the first 160 ms of inhalation would the aerosol reach the deeper airways. Thus, the time between the onset of inhalation and aerosol release, respectively, its inhalation is very time-critical. To minimize this time delay in the future, the aerosol valve should not only open when inhalation is detected, but at best shortly before. Although this means accepting possible losses due to aerosol release during the exhalation phase, the advantage of providing aerosol directly at the start of inhalation outweighs this in order to enable a higher aerosol deposition in the deeper airways. However, with precise timing of aerosol valve opening, the aerosol loss should be close to zero, with the highest possible aerosol deposition in the deeper airways. Even if the closing of the aerosol valve is not as time-critical as the opening of the aerosol valve, this parameter must still not be neglected—only if the aerosol valve closes according to the set parameters can a reduction of the aerosol loss or an accurate aerosol targeting be achieved.

The measured total aerosol valve opening duration, based on the duration of the aerosol detection, is 276.5 ± 9.3 ms for mode 1 and 409.5 ± 7.5 ms for mode 2. The set aerosol valve opening durations for the two breath-triggered drug release modes were 230 ms (mode 1) and 460 ms (mode 2). Thus, the set and measured values agree well for both modes. Due to the fact that the duration of the set aerosol valve opening corresponds to the duration of the aerosol detection, it can be stated that defined aerosol boluses can be applied. Thus, depending on the temporal release of the aerosol, the upper or the deeper airways can be targeted. By reducing deposition in the upper airways, a larger portion of the released aerosol dose can reach the lungs and thus the deeper airways [45,46,47,48,49,50]. This eventually leads to a lower variability of the drug dose in the lung [14]. The successful release of the aerosol boluses is also reflected in the calculated efficiency values *E_Valve_*, which are higher by a factor of 3.7 (mode 1) and 4.1 (mode 2), respectively, compared to the non-breath-triggered drug release.

Furthermore, in Figure 7a,b, a narrow-band flow and aerosol detection peak can be seen shortly after the aerosol valve opens. This is due to an increase in pressure upstream of the closed aerosol valve. As a result of the rapid opening of the aerosol valve, the compressed aerosol volume rapidly escapes, which is detected by the flow sensor and optically. This can be seen in particular in mode 1 (Figure 7a), since the aerosol valve remains closed longer compared to mode 2 (Figure 7b) and the pressure increase in front of the aerosol valve is therefore higher. Subsequently, the measured flow and the detected aerosol amount decrease rapidly again, which means a reversal of the direction of the aerosol flow and a short-term negative pressure, respectively. Due to the high rebound elasticity of the aerosol valve’s silicone membrane and the brief pressure pulse (overpressure) when the aerosol valve opens, the silicone membrane wall temporarily moves into the gas-filled functional space. Subsequently, the silicone membrane continues to oscillate as a damped spring-mass system due to its inertia. This is reflected in the flow measurement and in the optical detection of the aerosol by oscillations.

As mentioned before, aerosol reaches the alveoli only in the first half of the inspiratory phase [30], leading to an increased aerosol loss of up to 90% for the aerosol delivery during the entire respiratory phase in preterm neonates without triggered administration. This high loss corresponds well to previous reports about low depositions in infants [51,52,53,54,55,56], in some cases, less than 1% of the nominal dose [55,57]. Thus, breath-triggered drug release, especially for preterm neonates, is highly desirable. It is technically challenging, due to their high breathing frequency, short inspiration time and low tidal volume [17,18,54,58,59], but the results shown here could possibly be used to develop such devices. However, in addition, a trigger signal based on the detection of respiration is needed for a future application in the clinic. Possible systems for breath detection would be contact or non-contact systems [60,61,62,63,64,65,66,67,68]. When using a non-contact system, such as a time-of-flight camera [69,70], the preterm neonate is not affected nor is any interaction with the patient required [71]. In addition, flexible or stretchable sensor arrays, which can be attached to the skin, blanket or clothing, can also be used for breath detection [72,73]. This technology has already been successfully tested in experiments to generate trigger signals [74]. Thus, such systems can be used with a breath-triggered drug release system as described in this work. It is therefore up to the operator to decide which system to use for breath signal generation and whether the parameters of the selected system meet the required specifications.

Furthermore, the breath-triggered drug release system presented can be used independently of the medical substance, since the miniaturized valve triggers the release of the already generated aerosol. However, it must be taken into account that the pressure in the aerosol-conducting tube must be minimally greater than the pressure in the ventilation circuit respectively to the patient interface, otherwise no aerosol can be supplied to the patient through the valve. Thus, we have a slight pressure increase in the system, which, however, does not affect the ventilation system.

## 4. Conclusions

We showed that combining a miniaturized aerosol valve placed inside the patient interface for minimal distance with very short response time until full cross-section and breath-triggered drug release, we achieved a decreased loss of aerosol during exhalation, enhancing the efficiency up to a factor of 4.1 in comparison with continuous aerosol release. Thus, our system enables administration of high concentrations of pharmaceutical aerosol to (pre)term neonates during medical ventilation. The system also enables the delivery of targeted aerosol boluses at different inhalation times. In this way, the upper, deeper or entire respiratory tract can be targeted and fewer drugs need to be delivered to the body, resulting in a reduction in drug side effects. Costs can also be reduced considerably due to less aerosol loss and the aforementioned targeted therapy option. Therefore, this new approach of breath-triggered drug release in combination with any breath detection system, such as a time-of-flight camera, has great potential to be applied to (preterm) neonates in the future.

## Figures and Tables

**Table 1:** Summary of the three (breath-triggered) aerosol release modes.

Modus	Time of Aerosol Release after Detected Inhalation (ms)	Time of Aerosol Release Stop after Detected Inhalation (ms)	Total Aerosol Release Time (ms)
1	0	230	230
2	0	460	460
2	Continuous Aerosol Release		Cont.

## Data Availability

The data generated will be uploaded and made available in the research data repository of the Fraunhofer Society (Fordatis) (https://fordatis.fraunhofer.de, accessed on 24 April 2021).
